# The impact of milk suckling protocol and schedule on body weight and some morphometric measurements of Holstein heifers

**DOI:** 10.1007/s11250-022-03182-y

**Published:** 2022-05-11

**Authors:** Amr M. A. Rashad, Amani H. Amin, Ahmed E. Mahdy, Mahmoud A. Aziz, Adel S. A. El-Barbary, Dalia K. A. EL-Hedainy

**Affiliations:** Present Address: Animal and Fish Production Department, Faculty of Agriculture (Alshatby), Alexandria University, Alexandria El-Shatby, 11865 Egypt

**Keywords:** Suckling period, Holstein heifers, Performance, Body measurements, Daily gain

## Abstract

Having a successful heifer raising operation is not only relevant financially, but also influential for the future of the dairy herds. Milk feeding has a significant role on the health and growth of calves before weaning, in addition to the direct progressive effects on future performance post-weaning. Therefore, the aim of this study was to compare the effects of constants amount of milk (CMS) versus step-up/step-down (SUSD) on calf performance in two different suckling schedules of suckling milk gradually till 9th weeks of calf age, then decreasing it till weaning. For this study, forty Holstein heifers calves utilized between birth and 8 months of age were randomly divided into four groups of 10 calves each. Grouping was performed according to suckling protocol (constant versus variable) and suckling schedule (2 versus 3 times/day). Each calf suckled 600 kg of milk in a suckling period of 120 days. Body weight (BW), in addition to five body measurements: chest girth (CG), height at wither (HW), height at rump (HR), body length (BL), and diagonal length (DL), were recorded weekly. The heaviest BW was observed on SUSD calves suckling twice/day which had also the highest HW at weaning. Similarly, were BW, HW, and CG at 6 months of age. Also, the same group achieved the largest daily BW (0.902 kg/day), BL (0.246 cm), and CG (0.338 cm/day) gains during the pre-weaning phase. However, CMS suckled 3 times/day calves had the largest daily BW gain between birth and 6 or 8 months of age. CMS twice/day calves had the largest daily gain in CG from weaning to 6 months and from weaning to 8 months of age followed by SUSD suckling 3 times/day calves. Also, CMS twice/day calves had the largest daily gain in rump height from birth to 8 months of age compared to other groups. It can be concluded that when equal amounts of milk were fed during the suckling period, suckling protocol affected growth rate before weaning but that effect diminished as calf age increased after weaning.

## Introduction

Calf raising operations in most dairy farms are substantial and influential to the performance of future dairy cows. Having a successful calf raising operation is not only relevant financially, but is also important for the future success of the dairy herd (Cabral 2014). Suckling protocol is an expensive aspect in dairy farming. Starting calf heifers on sufficient balanced dieting is critical to their future performance, productivity, and longevity (Soberon et al. 2012). Implementing good health and caring strategies presents a main goal in calf heifers rearing protocols (Drackley 2008). Milk feeding has a significant role in the health and growth of calves before weaning, and has crucial influence on the post-weaning future performance (Khan et al. 2007b; Moallem et al. 2010). However, providing high amounts of milk does not necessarily improve performance, because of the challenges that arise during the suckling period such as reducing starter intake and consequently, impairment of rumen development (Khan et al. 2011). Dairy calves preweaning management regimes over the past decade has focused on utilization of alternative milk feeding methods in an attemptto improve calf growth performance and health without adverse effects on rumen development (Eckert et al. 2015; Omidi-Mirzaei et al. 2015). Omidi-Mirzaei et al. (2015) reported high average daily gain for calves fed milk in a step-up/step-down approach starting at 10%, increasing to 20% of body weight then decreasing gradually back down to 10% before weaning suggesting that the methods of milk delivery play a decisive role on calf performance. Moreover the amount of suckling milk may interact with frequency of suckling in artificially reared dairy calves. Also, to examine specific sucklings frequencies, several studies found no differences in weight gain of calves fed once or twice daily, when trails did not commence until the calves were 7 days or older (Kehoe et al. 2007; Gleeson et al. 2008). Furthermore, in cold climates, increasing the number of suckling from 2 to 3 times/day has improved weight gain and calf health (Schingoethe et al. 1986).

Therefore, the aim of this study was to compare the effect of constant amount of suckling milk against step-up/step-down suckling protocols under two suckling schedules: two and three times daily on calf performance.

## Materials and methods

This experiment was conducted on Holstein heifer calves raised in the Al-Alamia farm which belongs to Universal Company for Agricultural Development and Soil Reclamation located in Nubaria region 90 km south of Alexandria. All animals and experimental procedures in this study were supervised and approved by the Institutional Animal Care and Use Committee of Alexandria University, Egypt. Also, all procedures and experimental protocols were in accordance with the ethical standards as laid down in the 1964 Declaration of Helsinki and its later amendments.

### Animal and managements

For this study, 40 Holstein female calves with an average birth weight of 35 ± 2.66 kg were experimented between birth and 8 months of age, housed in individual pens in closed barn during the suckling period then moved to free open yards after weaning. All heifer calves were immediately separated from their dams at birth then fed on colostrum for 3 days before starting the suckling experiment. Calves were randomly divided into 4 groups of 10 calves each. All calves received the same starter and roughages ad libitum. They received 18% protein starter for 4 months then transferred to 16% protein starter until the end of the experiment. Starter feed was offered ad libitum until weaning; then, heifers received their requirements according to NRC 2001. Water was accessible all times, and routine vaccination program was followed regularly. Treatment groups were as follows:

1. constant amounts of milk were suckled equally three times/day (CMS3).
2. constant amounts of milk were suckled equally twice/day (CMS2).
3. step-up/step-down amounts of milk were suckled equally three times/day (SUSD3).
4. step-up/step-down amounts of milk were suckled equally twice/day (SUSD2).

### Experimental procedure

This experiment was conducted to evaluate the effect of suckling program: constant milk suckling (CMS) versus step-up/step-down (SUSD) and suckling schedule (2, at 6 a.m. and 6 p.m. versus 3, at 6 a.m., 1 p.m. and 8 p.m. sucklings/day) on Holstein female calves performance. SUSD suckling procedure aimed to provide greater amounts of milk to calves with the aim of encouraging it to consume extra starter ration to promote early rumen development. In this method, the amount of milk feeding increased gradually to reach a peak in the middle of the milk-feeding period before it is decreased gradually to the starting level immediately before weaning. Table 1 presents details of SUSD criterion of suckling protocol. In CMS, fixed amount of 5 kg of milk/day were offered to each calf throughout the whole suckling period (120 days). All calves eventually received the same amount of milk (600 kg in 120 days) right through the suckling period.

**Table 1:** The step-up/step-down suckling protocol criterion offered to each calf during suckling period

Age (days)	Period (days)	Kg of milk	Periodical milk, kg	Accumulated milk, kg
1–10	10	3	30	30
11–24	14	4	56	86
25–38	14	5	70	156
39–53	15	6	90	246
54–67	14	8	112	358
68–82	15	6	90	448
83–96	14	5	70	518
97–110	14	4	56	574
111–117	7	3	21	595
118–119	2	2	4	599
120	1	1	1	600

### Traits under study

**Fig. 1:**
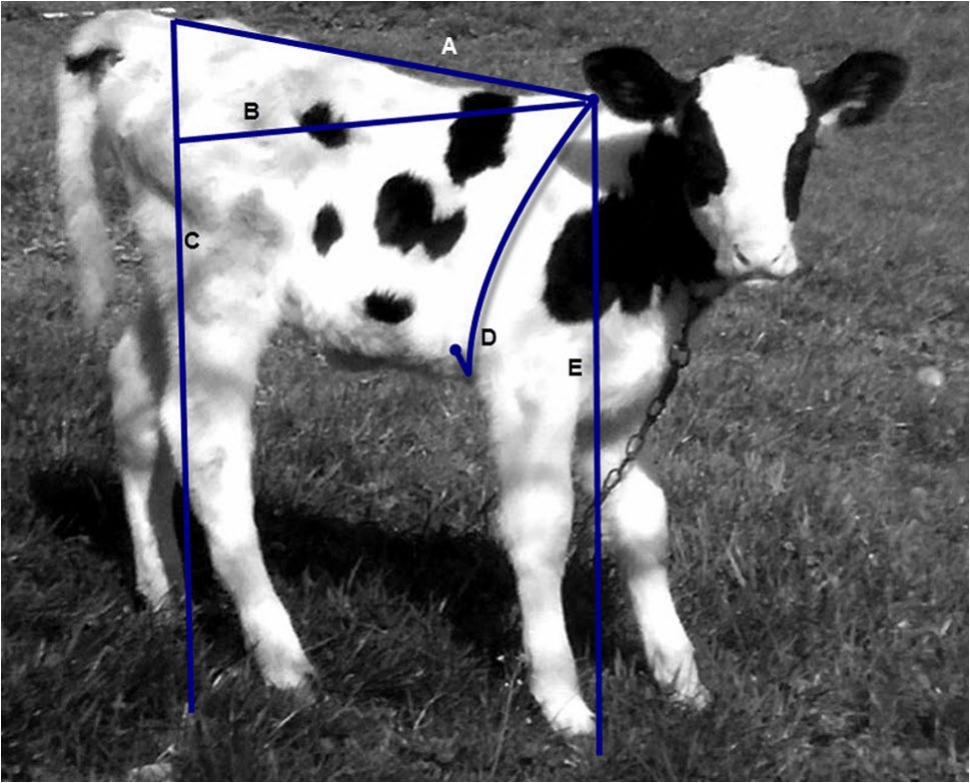
Body measurements taken on individual calves under study. A: Body length, was measured from the dorsal base of the head to the base of tail. B: Diagonal length was measured from the dorsal base of the head to the pin bone. C: Rump height was measured vertically from the rump to the ground. D: Chest girth was measured as the circumference behind the wither and shoulders. E: Wither height was measured vertically from the withers to the ground

All calves were weighed weekly from birth to weaning then monthly till the end of the experiment (8 months). Body weight (BW) was taken after fasting overnight using a hanged scale, to the nearest 0.5 kg. Immediately after weighing, five body measurements were recorded in centimeters (cm): chest girth (CG, was measured as the circumference behind the wither and shoulders), height at withers (HW, was measured vertically from the withers to the ground), height at rump (HR, was measured vertically from the rump to the ground), body length (BD, was measured from the dorsal base of the head to the base of tail), and diagonal body length (DL, was measured from the dorsal base of the head to the pin bone) as shown in Fig. 1. The height measurements were taken using a graduated measuring stick. The length and circumference measurements were measured using a tape ruler. All measurements were taken by the same technician to avoid personal error. Also, total gain and daily gain in body weight and measurements were calculated as Total gain = the current minus preceding calf body weight or measure and Daily gain = total gain divided by elapsed time (days). Also, concentrate feed intake was recorded individually during the suckling phase and the feed conversion was calculated for that period as follows: Feed conversion = Daily Feed intake, kg/Daily gain, kg.

### Statistical analysis

The data of the experiment were tested for normality using Shapire-Wilk test (*W* > 90), and were found normally distributed, then were statistically analyzed using GLM procedure of SAS (2009), and Duncan multiple range test was used to compare among means at 4 (weaning), 6, and 8 months of age if significance differences (*P* < 0.05) were observed. The following model was used to describe an observation: *Y*_*ijk*_ = *µ* + *S*_*i*_ + *T*_*j*_ + (S*T)_*ij*_ + *e*_*ijk*_, where *Y*_*ijk*_ = an observation on each studied trait, *µ* = the overall mean, *S*_*i*_ = the fixed effect of *i*^nd^ suckling program, *T*_*j*_ = the fixed effect of *k*^nd^ suckling schedule, (S*T)_*ij*_ = the interaction effect between suckling protocol and suckling schedule, and *e*_*ijk*_ = the random error terms.

## Results

All milk offered to all calves of different groups was consumed. No calf casualties occurred. Least squares means of BW and body measurements for calves at different ages in different treatment groups are shown in Table 2. The heaviest BW (157.6 kg) was observed on SUSD2 calves which had also the highest HW (106.6 cm) at weaning (*P* = 0.004 and 0.010, respectively). Similarly, the heaviest BW (197.4 kg), the highest HW (112.6 cm), and CG (130.0 cm) at 6 months of age were observed on SUSD2 calves. However, CMS2 calves had the smallest body measurements at the respective ages (*P* = 0.001, 0.004, and 0.049, respectively). In contrast, at the age of 8 months, heifers which suckled CMS3 achieved the largest average BW and BL (274.7 kg and 109.4 cm, respectively) compared to other groups (*P* = 0.030 and 0.040, respectively). Differences in other measurements were not significant. In general, the average of BW of SUSD groups at weaning was larger (*P* = 0.001) than that of CMS groups (148.3 vs. 109.5). Similarly, DL and HW at weaning of SUSD groups were larger than those of CMS groups. The same trend was observed for BW, DL, and CG at 6 months age, whereas those of SUSD calves were larger than those of CMS calves (*P* = 0.049, 0.001, and 0.040, respectively). At 8 months of age, SUSD calves were larger in BL, DL, and CG than the CMS groups. Besides, at 6 months age, three times suckling calves were heavier (*P* = 0.049) than the two times suckling groups (192.7 vs. 177.4).

**Table 2:** Least squares means of body weight and some body measurements of Holstein heifer calves at different ages, as affected by the interaction between suckling schedule (SS) and suckling protocol (SP)

Suckling schedules/d^1^	2	2	3	3	SEM		*P* value	
Suckling protocol^2^	CMS	SUSD	CMS	SUSD		SS*SP	SS	SP
At weaning (4 mo):								
Body weight, kg	100.5d	157.6a	118.4c	136.8b	5.5	0.004	0.800	0.001
Body length, cm	81.2	87.2	86.2	82.3	3.3	0.160	0.980	0.760
Diagonal length, cm	77.0	87.8	80.6	86.2	1.7	0.160	0.570	0.001
Wither height, cm	98.0c	106.6a	104.2ab	102.9b	1.7	0.010	0.490	0.049
Rump height, cm	108.2	114.9	111.6	112.2	2.3	0.210	0.890	0.140
Chest girth, cm	110.4	120.8	115.4	116.1	2.7	0.100	0.950	0.060
At 6 months:								
Body weight, kg	157.5c	197.4a	197.8a	187.0b	7.3	0.001	0.049	0.049
Body length, cm	91.1	92.0	97.8	89.3	3.5	0.180	0.560	0.290
Diagonal length, cm	84.7	95.5	88.6	94.2	1.3	0.060	0.330	0.001
Wither height, cm	105.5c	112.6a	110.4ab	108.0bc	1.6	0.004	0.910	0.130
Rump height, cm	115.1	120.0	118.1	118.2	1.4	0.100	0.660	0.080
Chest girth, cm	122.9c	130.0a	127.1b	127.1b	1.7	0.049	0.710	0.040
At 8 months:								
Body weight, kg	225.1d	260.2b	274.7a	247.3c	14.0	0.030	0.200	0.780
Body length, cm	107.0ab	104.4b	109.4a	98.0c	2.0	0.040	0.340	0.001
Diagonal length, cm	97.3	105.6	98.3	103.1	2.5	0.490	0.770	0.010
Wither height, cm	114.0	120.0	116.8	115.0	2.8	0.160	0.680	0.460
Rump height, cm	124.4	126.7	124.6	125.9	1.4	0.710	0.830	0.200
Chest girth, cm	138.0	141.1	135.0	140.9	2.0	0.480	0.440	0.020

^1^Suckling schedule: 2 times or 3 times sucklings/day

^2^CMS: constant milk suckling, SUSD: step-up/step-down suckling protocols

^a-d^Means with different letters within the same row are differ (*P*<0.05)

Least squares means of daily feed intake during the suckling period, feed conversion, and daily gain of BW and body measurements of heifers from birth to different ages of different treatment groups are presented in Table 3. During the suckling phase, SUSD2 calves recorded the largest feed intake (1.586 kg/day) followed by the SUSD3 calves (1.367 kg/day). However, the CMS3 calves achieved the lowest (best) feed conversion (1.536 kg intake / kg gain) during the suckling stage followed by SUSD3 (1.587 kg intake/kg gain). For the suckling phase, SUSD2 calves achieved the largest (*P* < 0.05) daily BW (902 g), BL (0.246 cm), and CG (0.338 cm) gains, but CMS2 calves had the smallest daily gain for the same respective traits (542 g, 0.202, and 0.256 cm). However, CMS3 calves had the largest daily gain of HW followed by SUSD2 calves (0.221 and 0.217 cm, respectively). From birth to 6 months of age, CMS3 group had the largest (*P* = 0.02) daily BW gain (907 g), while CMS2 calves had the lowest (679 g). SUSD2 and CMS3 calves had about the same daily gain of HW from birth to 6 months of age (0.183 and 0.182, respectively), but the CMS2 group had the smallest (0.159 cm). However, from birth to 8 months of age, CMS3 group had the largest (*P* = 0.04) daily BW gain (1.001 kg) followed by the SUSD2 group (0.931 kg), while the CMS2 calves had the smallest (0.791 kg). No differences were found among the 4 suckling protocol groups regarding daily gains of all body measurements from birth to 8 months age. In general, SUSD groups achieved the highest daily BW gain (882 g/day) and daily CG gain (0.332 cm/day) compared to CMS groups at suckling phase. However, no differences were observed between the two suckling protocols in daily gain of BW or body measurements from birth to 6 or 8 months of age except daily gain of CG (0.283 vs. 0.261 and 0.264 vs. 0.244 from birth to 6 and 8 months of age, respectively) and daily gain of BL (0.179 vs. 0.215) from birth to 8 months of age. In addition, 3 times suckling/day groups had larger daily BW gain from birth to weaning and from birth to 6 months of age (801 vs. 722 and 880 vs. 786 g, respectively) and daily CG gain (0.327 vs. 0.297 and 0.280 vs. 0.264 cm, respectively) from birth to weaning and from birth to 6 months of age compared to 2 times suckling/day groups. However, no differences were observed for all daily gains calculated for the two suckling schedule from birth to 8 months of age.

**Table 3:** Least squares means of daily feed intake (DFI) during suckling period, feed conversion, and daily gain of body weight and some body measurements of Holstein heifer calves from birth to different ages, as affected by the interaction between suckling schedule (SS) and suckling protocol (SP)

Suckling schedules/d^1^	2	2	3	3	SEM	*P* value		
Suckling protocol^2^	CMS	SUSD	CMS	SUSD		SS*SP	SS	SP
DFI during suckling period, kg	1.036d	1.586a	1.148c	1.367b	0.211	0.035	0.014	0.509
Feed conversion	1.911a	1.758b	1.536c	1.587c	0.147	0.019	0.022	0.542
Daily gain from birth to Weaning (4 mo):								
Body weight, kg/d	0.542d	0.902a	0.747c	0.861b	0.036	0.001	0.030	0.001
Body length, cm/d	0.202c	0.246a	0.246a	0.212b	0.017	0.020	0.760	0.760
Diagonal length, cm/d	0.217	0.236	0.244	0.234	0.012	0.400	0.340	0.700
Wither height, cm/d	0.178d	0.217b	0.221a	0.194c	0.009	0.001	0.240	0.510
Rump height, cm/d	0.192	0.225	0.225	0.222	0.009	0.060	0.100	0.110
Chest girth, cm/d	0.256c	0.338a	0.328b	0.326b	0.012	0.001	0.010	0.002
6 months:								
Body weight, kg/d	0.679d	0.893b	0.907a	0.851c	0.041	0.002	0.030	0.060
Body length, cm/d	0.193	0.178	0.227	0.184	0.019	0.450	0.310	0.140
Diagonal length, cm/d	0.193	0.208	0.206	0.200	0.009	0.270	0.640	0.740
Wither height, cm/d	0.159c	0.183a	0.182a	0.164b	0.009	0.020	0.780	0.710
Rump height, cm/d	0.171	0.186	0.184	0.187	0.007	0.430	0.310	0.220
Chest girth, cm/d	0.246	0.282	0.276	0.285	0.008	0.090	0.049	0.010
8 months:								
Body weight, kg/d	0.791d	0.931b	1.001a	0.889c	0.059	0.040	0.160	0.810
Body length, cm/d	0.211	0.183	0.218	0.174	0.009	0.380	0.920	0.001
Diagonal length, cm/d	0.197	0.198	0.195	0.188	0.012	0.770	0.590	0.740
Wither height, cm/d	0.155	0.168	0.164	0.152	0.012	0.320	0.770	0.930
Rump height, cm/d	0.167	0.167	0.165	0.173	0.007	0.600	0.820	0.590
Chest girth, cm/d	0.247	0.258	0.240	0.271	0.007	0.170	0.670	0.006

^1^Suckling schedule: 2 times or 3 times sucklings/day

^2^CMS: constant milk suckling, SUSD: step-up/step-down suckling system protocols

^a-d^Means with different letters within the same row are differ (*P*<0.05)

The interaction between suckling schedule and suckling protocol for daily gain of the studied traits from weaning to 6 and 8 months of age are shown in Table 4. CMS2 calves had the largest (*P* = 0.001) daily gain of CG from weaning to 6 months (0.202 cm) and from weaning to 8 months of age (0.226 cm) followed by SUSD3 group (0.170 and 0.200 cm, respectively). Also, CMS2 group had the largest (*P* = 0.030) daily gain of rump height (0.132 cm) from birth to 8 months of age compared to other groups. No differences were found for other traits. In general, no differences were observed for the effects of suckling schedule from weaning to 6 or 8 months of age, but suckling protocol affected (*P* < 0.05) daily BW and daily BL gains from weaning to 6 months of age. CMS groups achieved the best daily BW and daily BL gains (1.025 vs. 0.765 kg, 0.159 vs. 0.060 cm). The same trend was observed for daily gain of BL only from weaning to 8 months of age (0.194 vs. 0.107) compared to SUSD. No other differences were observed between suckling protocols.

**Table 4:** Least squares means of daily gain of body weight and some body measurements of Holstein heifer calves from weaning to different ages, as affected by of the interaction between suckling schedule (SS) and suckling protocol (SP)

Suckling schedules/d^1^	2	2	3	3	SEM	*P* value		
Suckling protocol^2^	CMS	SUSD	CMS	SUSD		SS*SP	SS	SP
Daily gain from weaning to:								
6 months:								
Body weight, kg/d	0.900	0.784	1.151	0.744	0.109	0.190	0.340	0.020
Body length, cm/d	0.154	0.018	0.164	0.106	0.036	0.280	0.180	0.010
Diagonal length, cm/d	0.124	0.126	0.107	0.110	0.023	0.990	0.490	0.900
Wither height, cm/d	0.103	0.093	0.081	0.086	0.017	0.680	0.400	0.880
Rump height, cm/d	0.111	0.084	0.079	0.094	0.013	0.120	0.420	0.680
Chest girth, cm/d	0.202a	0.138c	0.141c	0.170b	0.013	0.001	0.280	0.200
8 months:								
Body weight, kg/d	1.014	0.915	1.216	0.875	0.114	0.300	0.480	0.060
Body length, cm/d	0.210	0.089	0.179	0.125	0.018	0.080	0.890	0.001
Diagonal length, cm/d	0.167	0.147	0.134	0.130	0.024	0.760	0.300	0.620
Wither height, cm/d	0.123	0.109	0.094	0.101	0.021	0.620	0.390	0.850
Rump height, cm/d	0.132a	0.098c	0.093c	0.111b	0.012	0.030	0.280	0.520
Chest girth, cm/d	0.226a	0.161c	0.137c	0.200b	0.013	0.001	0.070	0.960

^1^Suckling schedule: 2 times or 3 times sucklings/day

^2^CMS: constant milk suckling, SUSD: step-up/step-down suckling system protocols

^a-c^Means with different letters within  the same row  are  differ (*P*<0.05)

## Discussions

SUSD suckling protocol accomplished the highest BW and HW throughout the experiment up to weaning. Also, this protocol of suckling produced the highest daily BW gain during suckling phase with the majority reporting benefits in terms of daily BW, BL, and CG gains during the pre-weaning period. Such surpass was probably due to regulating the amount of suckled milk according to the calf requirements. In contrast, CMS3 heifers achieved the best performance after weaning till 8 months of age probably due to that partitioning the calf share into three sucklings should improve feeding efficiency and allow for low (good) feed conversion during suckling phase which consequently improved the heifer performance when turned to solid feeding. Similarly, Hosseini et al. (2019) reported that calves fed SUSD milk were heavier at weaning and had greater BL, HW, CG, and RH. The same tendency was observed by Quigley et al. (2018). Moreover, Khan et al. (2007b) clarified that gradual decrease of suckling milk toward weaning could implicate the mitigation of the adverse effects on the post-weaning growth when feeding high amount of milk pre-weaning. The amount and schedule of milk feeding are influential on growth and development of artificially reared calves. However, most artificial calf-rearing protocols are economically driven to minimize milk suckling costs via restricting milk allowances by weaning calves earlier. Calves receiving greater amounts of milk (Khan et al. 2011) or weaned late exhibited improved growth performance at weaning (Sweeney et al. 2010; Eckert et al. 2015), but, solid feed intake was negatively correlated with the amount of milk supplied to calves (Khan et al. 2011).

Comparisons have been made to evaluate the effect of increasing milk feeding during suckling phase against the early consumption of solid starter rations which might speedup the rumen development of dairy calves (Brown et al. 2005; Silper et al. 2014). However controversial results have been reported regarding the performance of young calves fed large amounts of milk before weaning compared to those on conventional milk-feeding protocols. Although some studies have reported improvements in calves growth rates (Huuskonen and Khalili 2008), feed efficiency, BW gain, and performance (Diaz et al. 2001; Khan et al. 2011; Silper et al. 2014), others have shown that feeding high amounts of milk to young calves could reduce the starter intake, growth, and reticulorumen development (Baldwin et al. 2004; Terré et al. 2007). The discrepancies observed may be due to differences in the amounts, quality, and methods of feeding the liquid feed (milk or milk replacer).

Many researches have focused on alternative procedures of milk feeding to dairy calves attempting to improve weight gain and general health without adverse effects on starter consumption or ruminal development. In conventional milk feeding, calves receive restricted amounts of milk or milk replacer (4 L/day, equivalent to approximately 10% of birth weight) during the whole suckling period (Khan et al. 2007a,b; Silper et al. 2014). According to Davis and Drackley (1998), the conventional milk feeding method resulted in decreased alimentary efficiency, thereby leading to suboptimal performance of dairy calves during the whole milk-feeding period. Alternatively, the step-down milk-feeding procedure allows calves to receive greater amounts of milk during the early weeks of the suckling period (6 L/day milk from day 1 to 29), followed by a fixed amount of milk until weaning (4 L/day milk from day 30 to 60). Khan et al. (2007b) showed that using the step-down method rather than the conventional procedure of milk feeding not only improved BW gain and feed efficiency but also prevented the problems associated with depressed solid feed intake. Moreover, Silper et al. (2014) reported similar performance on calves fed milk replacer via both procedures.

However, limited, if any, evidence is available on the influence of SUSD procedure on the performance of dairy calves. In a previous study, Terré et al. (2007) found that starter dry matter intake decreased during the preweaning and postweaning phases when milk was fed to calves in the enhanced-growth feeding program that consisted of 4 L/day from day 1 to 6, 6 L/day from day 7 to 13, 7 L/day from day 14 to 20, 6 L/day from day 21 to 28, and 3 L/day from day 29 to 35. In that study, the calves under the program exhibited lower nutrient digestibility coefficients in the week after weaning compared to conventionally fed calves.

In the present study, 3 times sucklings per day achieved the best calves’ performance of BW at 6 months of age and daily gain at weaning and 6 months of age as compared to twice sucklings daily, which confirmed that dividing the calf daily milk allowance into three meals would improve digestability and efficiency of milk utilization by the calf. In contrast, Saldana et al. (2019) detected no differences between once or twice-a-day feeding protocol for BW (*P* = 0.17), HW (*P* = 0.32), and RH. Similarly, Kienitz et al. (2017) and Stanley et al. (2002) found that feeding dairy calves milk or milk replacer once or twice daily had no adverse effects on BW, weight gain, RH, or CG which all were not different between feeding groups. Also, Kehoe et al. (2007) found that calves did not differ in growth measurements whether they were fed once or twice daily.

## Conclusion

When equal quantities of milk were fed to dairy calves during the suckling period, the protocol, and times of milk feeding affected growth rate before weaning. This effect was diminished by progress of calf age after weaning. SUSD suckling protocol in general achieved the highest BW, body measurements, and daily BW gain up to weaning compared to CMS protocol. But CMS3 protocol achieved the best heifers’ performance after weaning till 8 months of age.

## Data Availability

Not applicable.
